# Histone demethylase JMJD2C: epigenetic regulators in tumors

**DOI:** 10.18632/oncotarget.19176

**Published:** 2017-07-12

**Authors:** Chengcheng Zhang, Zhongqi Wang, Qing Ji, Qi Li

**Affiliations:** ^1^ Department of Medical Oncology, Shuguang Hospital, Shanghai University of Traditional Chinese Medicine, Shanghai 201203, China; ^2^ Department of Medical Oncology, Longhua Hospital, Shanghai University of Traditional Chinese Medicine, Shanghai 200032, China

**Keywords:** histone demethylase, JMJD2C, epigenetic regulation, tumor

## Abstract

Histone methylation is one of the major epigenetic modifications, and various histone methylases and demethylases participate in the epigenetic regulating. JMJD2C has been recently identified as one of the histone lysine demethylases. As one member of the Jumonji-C histone demethylase family, JMJD2C has the ability to demethylate tri- or di-methylated histone 3 and 2 in either K9 (lysine residue 9) or K36 (lysine residue 36) sites by an oxidative reaction, thereby affecting heterochromatin formation, genomic imprinting, X-chromosome inactivation, and transcriptional regulation of genes. JMJD2C was firstly found to involve in embryonic development and stem cell regulation. Afterwards, aberrant status of JMJD2C histone methylation was observed during the formation and development of various tumors, and it has been reported to play crucial roles in the progression of breast cancer, prostate carcinomas, osteosarcoma, blood neoplasms and so on, indicating that JMJD2C represents a promising anti-cancer target. In this review, we will focus on the research progress and prospect of JMJD2C in tumors, and provide abundant evidence for the functional application and therapeutic potential of targeting JMJD2C in tumors**.**

## INTRODUCTION

**Figure 1:**
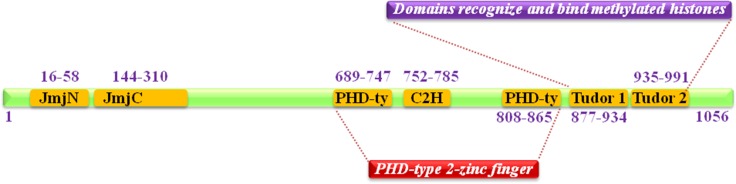
Schematic summary of JMJD2C domain structure It contains one JmjN domain (N-terminal Jumonji N domain), one JmjC domain, two PHD (C-terminal plant homeodomain) and two Tudor domains that recognize and bind methylated histones.

**Figure 2:**
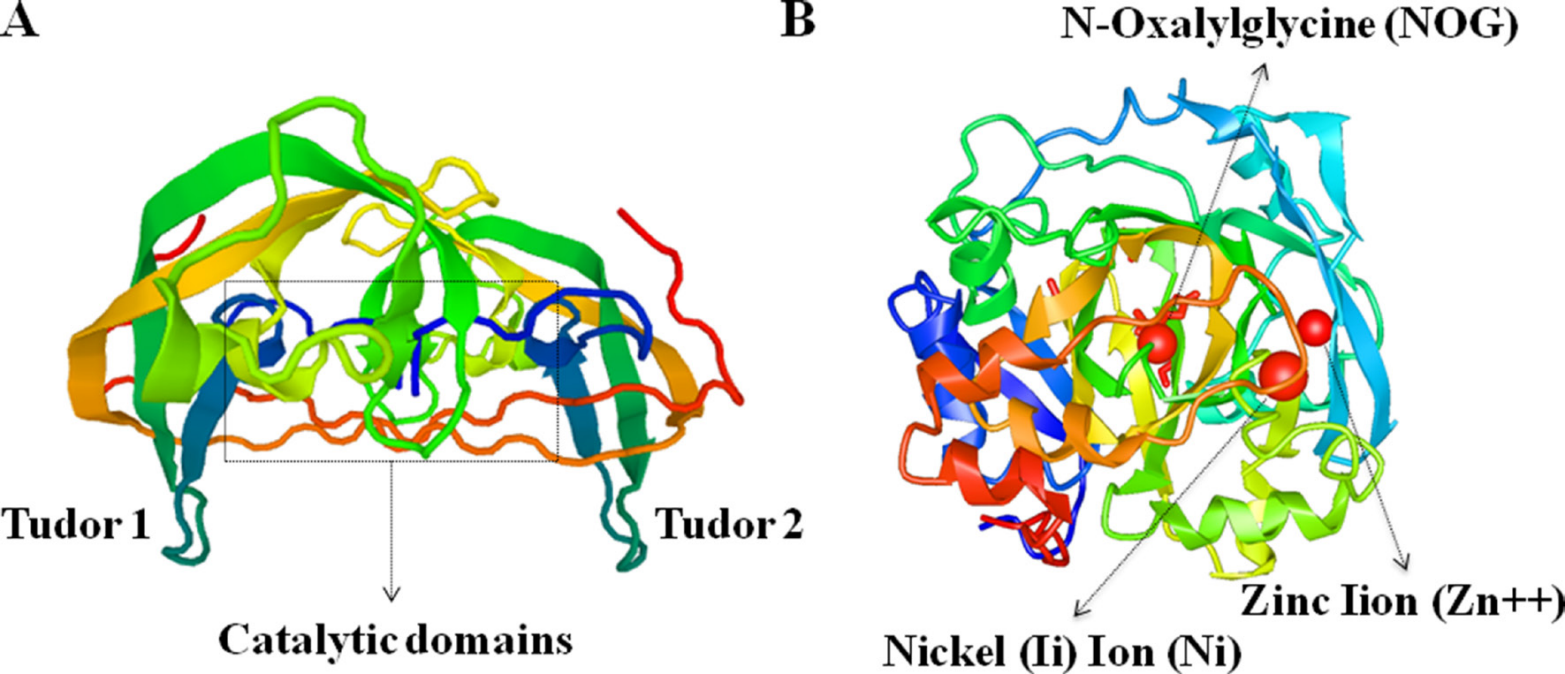
Crystal structure of human JMJD2C protein characterized by X-RAY diffraction (**A**) The tudor domains that recognize and bind methylated histones, and the catalytic domains are marked in the schematic image of JMJD2C crystal structures. (**B**) The functional moleculars including NOG (N-Oxalylglycine), Zn (Zinc Iion) and Ni (Nickel (Ii) Ion) are presented in the precise image of JMJD2C crystal structures.

JMJD2C (Jumonji domain 2C), also well known as KDM4C (Histone lysine demethylases 4C), is mapped to human chromosome 9p24.1, encoded a protein with 1054 amino acid residues. It is one member of Jumonji domain-2 families and encodes a protein with one JmjN domain (N-terminal Jumonji N domain), one JmjC domain, two PHD (C-terminal plant homeodomain) and two Tudor domains which mostly localized in the nucleus (Figure 1) [1, 2]. The crystal structure of the catalytic domains and tudor domains of human JMJD2C characterized by X-RAY diffraction was shown in Figure 2 [3–5].

JMJD2C has been proved to be a demethylase for H3K9 methylation, in the manner of catalyzing the demethylation of H3K9me3/me2 (the known repressive markers of gene regulation), a histone mark found in heterochromatin associated with euchromatic transcriptional silencing and heterochromatin formation [6]. Besides, JMJD2C also catalyzes the demethylation of H3K36me3 (the second methylated histone substrate of JMJD2C) [3]. In addition, JMJD2C has been reported to demethylates H3K9me3 by two sequential reactions: removing the methyl groups from methylated lysines of histone H3 and disconnecting from the nitrogen center [7].

JMJD2C has been shown to have a variety of function both in normal biology and in tumorigenesis. Early in year 2007, JMJD2C was firstly found to involve in embryonic development and stem cell regulation [8–11], and it acted as a positive regulator for Nanog (a key transcription factor for self-renewal in embryonic stem cells) by reversing the H3K9Me3 marks at the Nanog promoter region and consequently prevents transcriptional repressors HP1 (heterochromatin protein 1) and KAP1 (Krüppel-associated box (KRAB) domain-associated protein 1) from binding. JMJD2C was necessary for self-renewal of ESCs and induced pluripotent stem cell generation by assisting PRC2 (Polycomb repressive complex 2) in transcriptional repression [12]. JMJD2C was also found to regulate neural stem cell differentiation by controlling the gene-specific methylation of H3K9 and H3K36 on neurotrophic BDNF versus astroglial GFAP genes [13]. However, more and more evidences have also demonstrated that JMJD2C play an important role in the tumorigenic processes. JMJD2C could regulate androgen receptor-dependent gene expression, embryonic stem-cell self-renewal, and tumorigenesis [14]. Overexpression of JMJD2C could up-regulate the expression of oncogene Mdm2 (murine double minute 2) and lead to the decreased expression of tumor suppressor gene p53 [15]. Notably, as the downstream targets of BMP4, bFGF and VEGF, JMJD2C activated VE-cadherin expression and allowed the cells to form stable adherens junctions and functional vascular networks [16, 17]. All the reported results demonstrated that JMJD2C is a candidate oncogene.

In this review, we will focus on the research advance and future prospect of oncogene JMJD2C in tumors, and provide enough evidence for functional application and therapeutic potential of targeting JMJD2C in tumors.

## ORIGINS AND PROPERTIES OF JMJD2C

It is well known that, histone modifications can regulate gene expression by establishing global chromatin environments [18, 19, 20], and these epigenetic changes in chromatin environments are the underlying causes of tumors development. The amino-terminal tails of histone are subjected to numerous reversible posttranslational modifications, such as phosphorylation [21], acetylation [22], ubiquitination [23], sumoylation [24], ADP ribosylation [25], and methylation [26, 27]. Among them, methylation, can specifically modify chromatin structure and regulate gene expression [28, 29]. Histone methylation was considered as a stable modification until the discovery of lysine-specific demethylase 1 (LSD1) in 2004, which was the firstly identified histone demethylase. The discovery of LSD1 demonstrated that, histone methylation was reversible and dynamically regulated [30–33].

Currently, the histone demethylases are classified into two types. The first type of histone demethylase includes LSD1a (lysine specific demethylase 1) and LSD2 (lysine specific demethylase 2) [34]. The other type of histone demethylase comprises the recently discovered histone demethylases JMJD (Jumonji-C domain-containing protein) family [35]. The JMJD subfamily, comprising member A, member B, member C and member D, involves in the dynamic accommodation of histone posttranslational modification, controlling multiple biological processes such as gene transcription, epigenetic silencing, heterochromatin formation, and so on [36–38]. As we know, the lysine residues in histone tails could be mono-, di- or tri-methylated (me1/me2/me3), and the differentially methylation of lysines can result in different physiological and transcriptional outcomes. Additionally, the methylation status of H3K9 (Histone H3 lysine 9) was associated tightly with the affinity of heterochromatic proteins, which could affect the organization and stability of chromosome [39]. JMJD demethylases A, B, C and D were found to have the demethylation function on trimethylated lysines [40].

A few studies have revealed that JMJD2A is nearly comparable in the cytoplasm and nucleus, JMJD2B is mainly found in the nucleus, and JMJD2C is associated closely with the chromatin [41, 42]. More interestingly in colon cancer cells, JMJD2C appears to be co-overexpressed with JMJD2A and JMJD2B, and JMJD2C also forms heteromers with JMJD2A, whereas JMJD2D does not have the same function as JMJD2C [43]. Additionally, JMJD2C was also found to increase MyoD (Myogenic Differentiation Antigen) transcriptional activity and facilitate skeletal muscle differentiation via inhibiting G9a-dependent MyoD degradation [44].

## EPIGENETIC REGULATION OF JMJD2C IN TUMORS

**Figure 3:**
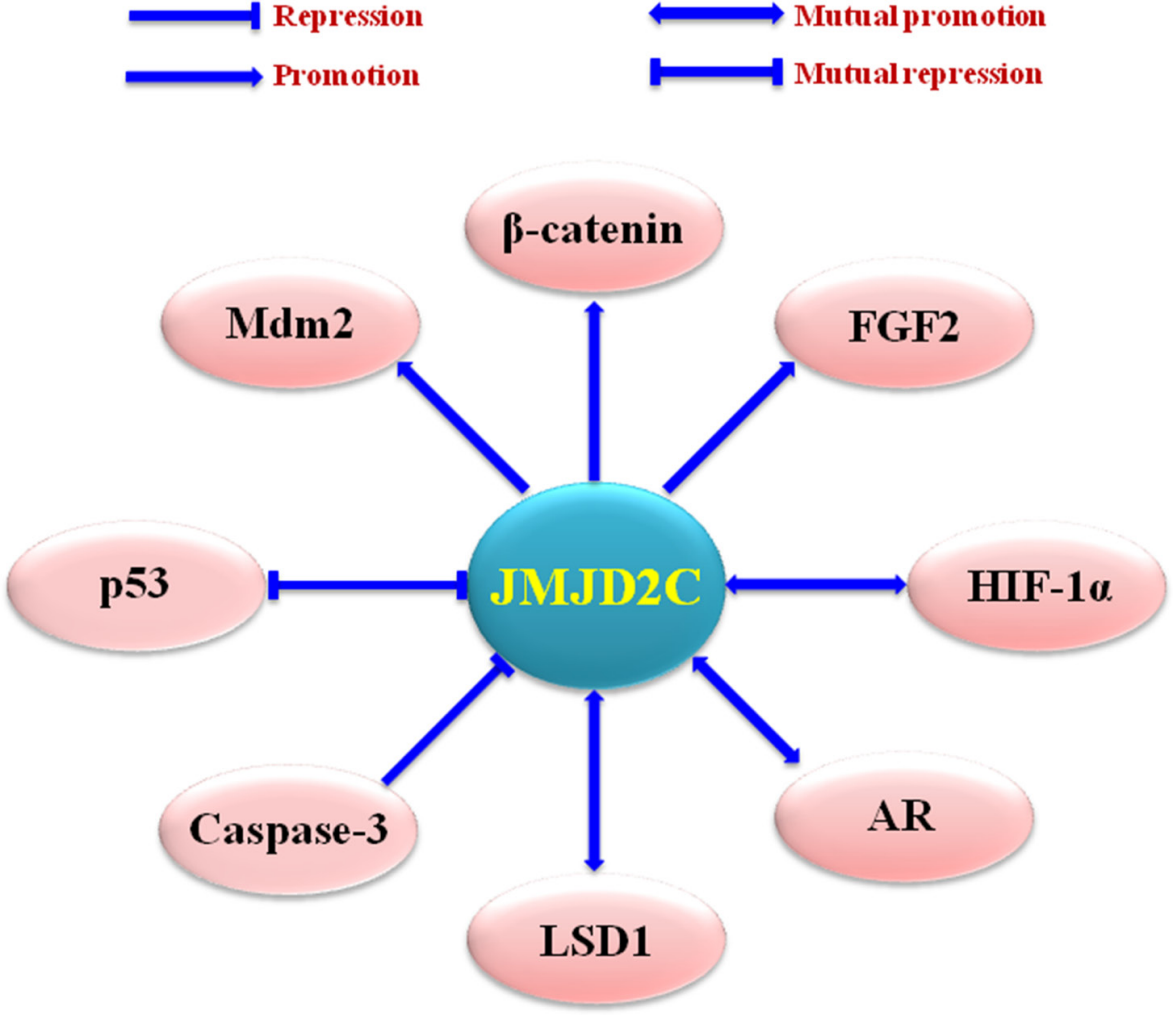
Upstream regulating factors and downstream effectors of JMJD2C Mdm2 (murine double minute 2), AR (androgen receptor), LSD1 (lysine-specific demethylase 1), and FGF2 (fibroblast growth factor 2).

Dysregulation of JMJD2C has been detected in a variety of tumors, including oesophageal squamous cell carcinoma (ESCC) [45], acute myeloid leukemia (AML) [46], primary mediastinal B cell lymphoma (PMBL), Hodgkin lymphoma (HL) [47], medulloblastoma [48], prostate cancer [49], and breast cancer [50, 39] (Table 1). In these tumors, JMJD2C could modulate the transcription factors such as androgen and estrogen receptor (AR and ER) [51], the nuclear receptor superfamily like glucocorticoid receptor (GR) and progesterone receptor (PR) [52, 53], the hypoxia-inducible factors such as HIF-1α (Hypoxia-inducible factor-1α) [54], and so on (Figure 3).

**Table 1:** Biological function of JMJD2C in tumors

Tumor types	Functions of JMJD2C	References
Breast cancer	Metabolic reprogramming	[39]
	Tumor cell growth and proliferation	[39, 58, 59]
	Lung metastasis	[39]
	Cellular transformation	[58, 59]
	DNA damage and repair	[55, 56, 57]
Prostate cancer	Tumor growth, proliferation and metastasis	[64, 65]
Colon cancer	Tumor growth and proliferation	[79, 80]
Osteosarcoma	Tumor proliferation, migration and invasion	[72]
Gastric cancer	Tumor growth, invasion and metastasis	[81]
Esophageal squamous cancer	Tumor cell growth	[45]
Sarcomas/Leukemias/Gliomas	Cancer development	[75, 76, 77]
Primary mediastinal B-cell lymphoma/Hodgkin lymphoma	Tumor formation	[47]
Lung cancer	Tumor cell migration and invasion	[82]

### Breast cancer

Recent evidences have implied that alteration of JMJD2C demethylases was associated closely with the progression of breast cancer [50, 55–57]. Hong et al. [55] has identified JMJD2C as a novel substrate for caspase-3 (cysteine-aspartic acid protease-3), and cleavage of JMJD2C by caspase-3 led to inactivation of JMJD2C demethylase activity and elevation of H3K9 methylation levels. JMJD2C could be cleaved by caspase-3 at DEVD396G motif ((between residue 475-525), and then its demethylase activity would inactivate. In addition, D396N polymorphism (rs2296067) in the cleavage site of JMJD2C influenced the cleavage by caspase-3, following by the affection on the prognosis of breast cancer. Moreover, Hong et al. also showed that, the basal levels of DSB (double strand DNA break) repair proteins γ-H2AX increased when the activity of caspase-3 was activated. Knockdown of JMJD2C gene led to the up-regulation of basal γ-H2AX expression. and γ-H2AX together with its phosphorylated C-terminal (Sre residues 139–140, γ-H2AX) are crucial for DNA repair [56, 57]. These findings provide a novel epigenetic regulating mechanism of JMJD2C by caspase-3 cleavage, and the single nucleotide polymorphism rs2296067 of JMJD2C alters the cleavage by caspase-3 and affect the prognosis of breast cancer.

Ye et al. previously demonstrated that, JMJD2C was overexpressed in aggressive basal-like breast cancer and functioned as oncogene. Afterwards, using a large-scale data set for cancer genomics, they validated that JMJD2C was high expressed in 12.4% of basal-like tumors, and significantly higher in aggressive basal-like breast cancer in compared with non-basal-like breast cancer. When JMJD2C was overexpressed in immortalized and nontransformed mammary epithelial MCF10A cells, it promoted growth factor-independent proliferation and anchorage-independent growth, as well as the alteration of morphogenesis in Matrigel and the mammosphere forming ability. Moreover, JMJD2C could regulate the expression of genes critical for stem cell self-renewal like NOTCH1, and was closely associated with the stem cell phenotypes in breast cancer [58]. In subsequent studies, Ye et al. also found JMJD2C served as a transforming oncogene. Knockdown of JMJD2C inhibited the proliferation of breast cancer cells *in vitro* and *in vivo*. More importantly, comparing with other members of JMJDs family, JMJD2C had the highest frequency (13.4%) of genetic alteration including mutation, homozygous deletion, high-level amplification, mRNA downregulation, and mRNA upregulation among 976 breast cancer specimens, and all the 976 Cancer Genome Atlas (TCGA) data for breast cancer were from cBio Cancer Genomics (http://cbioportal.org) database [59].

**Figure 4:**
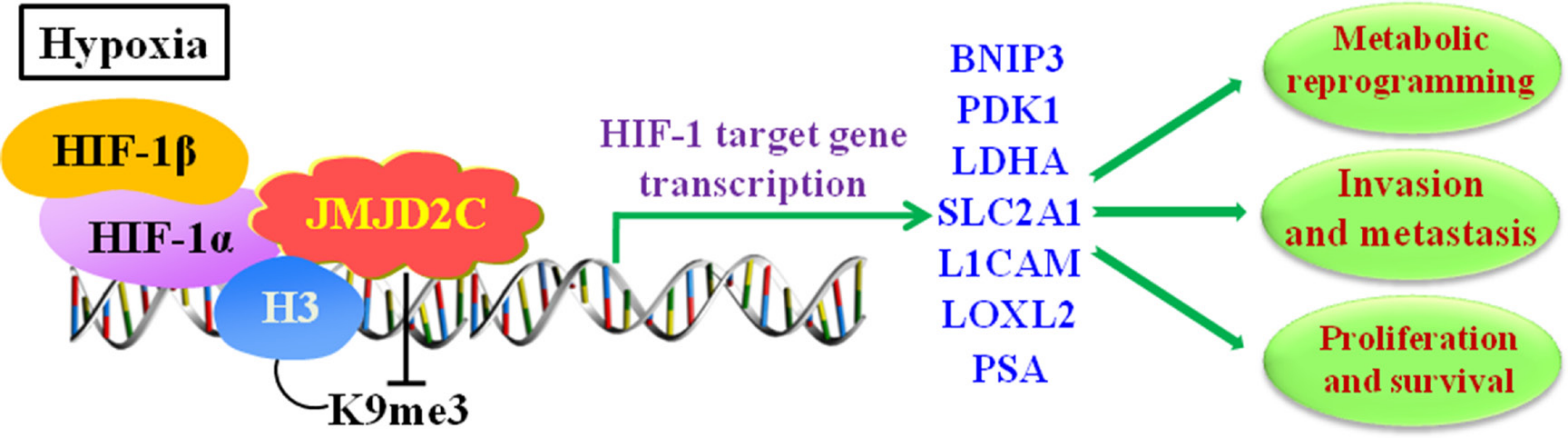
JMJD2C regulates the transcription of HIF-1 target gene in hypoxic environment, and involves in cancer progression including proliferation, invasion, metastasis, and metabolic reprogramming in tumors In hypoxia, HIF-1α dimerizes with HIF-1β to form active HIF-1 complex. JMJD2C interacts with HIF-1α and promotes the transcriptional activation of HIF-1 targeting genes via demethylating di- and trimethylated H3K9. The HIF-1 targeting genes include BNIP3 (BCL2 Interacting Protein 3), PDK1 (Pyruvate Dehydrogenase Kinase 1), LDHA (Lactate Dehydrogenase A), SLC2A1 (Solute Carrier Family 2 Member 1), L1CAM (L1 Cell Adhesion Molecule), LOXL2 (Lysyl Oxidase Like 2), and PSA (prostate-specific antigen).

Strikingly, the findings by Luo et al’s. suggested in breast cancer cells that [39], increased expression of JMJD2C activated the transcription of PDK1 (Pyruvate Dehydrogenase Kinase 1), LDHA (Lactate Dehydrogenase A), BNIP3 (BCL2 Interacting Protein 3) and SLC2A1 (Solute Carrier Family 2 Member 1), and all of their encoded proteins [60–63] were required for metabolic reprogramming thereby stimulating HIF-1-dependent reprogramming. Moreover, knockdown of JMJD2C not only inhibited the breast tumor growth but also blocked the lung metastasis in mice. To sum up, these results reveal the important epigenetic roles of JMJD2C in breast cancer that it regulates the metabolic reprogramming and lung metastasis by coactivating HIF-1 and stimulating HIF-1-mediated transactivation (Figure 4).

### Prostate cancer

Various studies have shown that, JMJD2C was overexpressed in prostate cancers and promoted abnormal activation of AR (androgen receptor) targeting genes such as kallikrein-related peptidase 2 (KLK2) and prostate-specific antigen (PSA), and both of which had the important function to cleave proteins that affecting cell proliferation and metastasis. In details, JMJD2C knockdown promoted the existence of H3K9me3 on the PSA gene, following by blocking AR-induced transcriptional activity [64].

Wissmann et al. [65] showed that, JMJD2C was found to be co-localized with AR and LSD1 in the epithelium of prostate carcinoma and normal prostate cells. For the detailed mechanism, JMJD2C, AR and LSD1 assembled on the chromatin to remove the methyl groups from mono-, di- and trimethylated H3K9. Importantly, JMJD2C specifically removed the demethylation of the trimethyl H3K9 marks and modulated the transcriptional activity of AR. Moreover, JMJD2C cooperated with LSD1 and activated AR-mediated gene expression via decreasing H3K9me3 at the promoter of AR targeting genes KLK2 and PSA. Accordingly, Hamada et al. [66] suggested that, combined application of JMJD2 inhibitors and LSD1 inhibitors would provide a novel strategy for cancer therapy.

Furthermore, Lee et al. also revealed that, in human LNCaP prostate cancer cells, the hypoxia situation increased the levels of JMJD2C protein, and the pathological hypoxia (< 0.5% O_2_) brought about the abnormal variations of epigenetic genes through modifying the quantity and activity of JMJD2C because of the existence of H3K9me3 (the substrate for JMJD2C). In details, JMJD2C has been identified as a HIF-1α targeting gene [67]. Endogenous JMJD2C has been found to physically interact with HIF-1α, which was involved in multi-steps of cancer progression, including cell proliferation, angiogenesis, invasion and metastasis, as well as the glucose and energy metabolism in human cancer cells [68–71]. To be concluded, these findings imply in prostate cancer that, JMJD2C interacts with HIF-1α and promotes the transcriptional activation of PSA gene via demethylating di- and trimethylated H3K9 (Figure 4).

### Osteosarcoma

Similar to above common cancers, Li et al. [72] also showed the expression of JMJD2C was significantly higher in osteosarcoma tissues in compared with adjacent non-tumor tissues. Besides, the high level of JMJD2C was strongly associated with osteosarcoma cells metastasis, indicating that JMJD2C acted as a risk factor of osteosarcoma. As far as the detailed mechanism in osteosarcoma, JMJD2C could up-regulate the expression of FGF2 (fibroblast growth factor 2) and interact with FGF2, which is a prototypic growth factor [73] and plays a promoting role in carcinogenesis [74]. The silence of JMJD2C gene significantly inhibited the osteosarcoma cell invasion and migration in MG63 cells. These results indicated that, JMJD2C could promote osteosarcoma cell proliferation, migration and invasion via interacting with FGF2.

### Blood neoplasms

Co-recruitment of JMJD2C and Prmt1 (protein arginine methyltransferase 1) by MOZ (monocytic leukemia zinc finger protein)-TIF2 (transcriptional intermediary factor 2) and MLL (malignant lymphoma with leukemia)-GAS7 (Growth arrest-specific 7) in acute promyelocytic leukemia (APL) has implied the possible mechanism of JMJD2C in oncogenesis. Specifically, JMJD2C counteracted and removed the H3K9 trimethylation on target gene Hoxa9, which was critical for oncogenic transformation and self-renewal [75]. In addition, the inhibition of JMJD2C suppressed Acute Myeloid Leukemia (AML) by suppressing transcription and transformation abilities of MLL fusions and MOZ-TIF2 [76]. Agger et al. also showed that, JMJD2/KDM4 demethylases (including JMJD2C) are required for the expression of Il3ra (interleukin 3 receptor subunit alpha), as well as the survival of acute myeloid leukemia cells [77].

JMJD2C was considered as an oncogene due to its firstly discovered in primary Hodgkin lymphoma and mediastinal B-cell lymphoma, in which both of JMJD2C and tyrosine kinase JAK2 (Janus kinase 2) were found to have the function of epigenetic regulation. Inhibition of both JMJD2C and JAK2 could kill these lymphomas by promoting heterochromatin formation through decreasing tyrosine 41 phosphorylation and increasing lysine 9 trimethylation of histone H3. c-Myc was the major target of JAK2-mediated histone phosphorylation. When JMJD2C and JAK2 were inhibited, c-Myc was corresponding silenced, following by the c-Myc locus to adopt a repressive heterochromatic structure. Moreover, JMJD2C knockdown or JAK2 inhibition increased the recruitment of heterochromatin protein HP1α to the c-Myc locus, as well as the increase of HP1α protein in H3K9me3, which is bound by HP1α. In conclusion, JMJD2C and JAK2 cooperatively remodel the epigenome of Hodgkin lymphoma and primary mediastinal B-cell lymphoma, providing the possibility of the discovery of JAK2 and JMJD2C inhibitors for these malignant diseases [47].

Moreover, the JMJD2C gene was also found to be translocated in mucosa-associated lymphoid tissue (MALT) lymphoma and led to rearrangements of ODZ2 (odd Oz/ten-m homolog 2), JMJD2C and CNN3 (Calponin-3), which broadened the knowledge on the genetic heterogeneity of MALT lymphomas [78].

### Other cancers

In colon cancer, Kim et al. firstly uncovered that JMJD2C was overexpressed in five colon cancer cell lines and especially associated closely with the growth of HCT-116 cells. Then they observed that transcriptional cofactor JMJD2C might form a complex with β-catenin, thereby regulating the levels of β-catenin downstream effectors, which was crucial for the advance of colon cancer. Furthermore, JMJD2C was required for efficient expression of cell cycle regulator Cyclin D1, oncogenic transcription factor FRA1 (Fos-related antigen 1), and anti-apoptotic BCL2 (B-cell lymphoma-2). These results strongly suggested that, JMJD2C might promote the survival of colon cancer cells via BCL2-dependent mechanism, and stimulate the proliferation of colon cancer cells via up-regulating the levels of Cyclin D1 and FRA1 [79]. In addition, Kim et al. also demonstrated that, down-regulation of JMJD2C led to the increased levels of tumor suppressor p53 [80].

Xu et al. explored the expression of JMJD2C in gastric carcinoma using a retrospective cohort study for 110 gastric cancer patients. As a result, the positive expression rates of JMJD2C were significantly higher in tissues from gastric cancer patients than those in normal tissues. Besides, JMJD2C expression was positively correlated with the HIF-1α expression. Over-expressions of JMJD2C and HIF-1α in gastric cancer tissues were associated closely with the growth, invasion and metastasis of gastric tumor, implying that JMJD2C might be a bad prognosis marker for gastric cancer patients [81]. Recently, Li et al. also found that, JMJD2C could promote cell migration and invasion through modulating the CUL4A expression in lung cancer [82].

Although above reports have demonstrated the significance of JMJD2C in tumors, to be an effective therapeutic target, further studies are still needed to explore clearly the underlying mechanisms of JMJD2C in tumors.

## THERAPEUTIC POTENTIAL OF JMJD2C

Since epigenetic changes are reversible, the histone demethylase JMJD2C is a promising therapeutic target for different tumors [59]. By far, several JMJD2C inhibitors have been developed. N-oxalylglycine 2 (NOG2), the amide analogue of α-ketoglutarate, has been reported to inhibit the activity of JMJD2C proteins *in vitro* [83]. Tan et al. also demonstrated the catalytic site of JMJD2C could be inhibited by the competitive antagonists of α-ketoglutarate [84], providing that JMJD2C might be a novel target for the treatment of breast cancer. Moreover, as one of the series of hydroxamate analogues designed by Hamada et al. hydroxamate analogue 8, showed 9100-fold greater JMJD2C-selectivity and 500-fold greater JMJD2C-inhibitory activity in compared with the NOG2 and other inhibitors for demethylase members [66, 85].

Additionally, Qin et al. has tested the effect of small molecule NCDM-32B, a novel inhibitor of JMJD2 demethylases, which had the function to attenuate the growth of breast cancer cells [59]. The NCDM-32B treatment inhibited the cell viability and anchoraged independent growth in breast cancer, and the mechanism referred to several vital signaling pathways that promote cell proliferation and transformation.

Besides, an unexpected function of JMJD2C was found in the DNA damage pathway. Radiation and chemotherapy may cause DNA damage and induce signaling pathways for apoptotic cell death. DNA repair processes play key roles in the resistance to chemotherapy and radiation therapy, and H3K9me3 is involved in the initial processing of DNA repair [86]. The cleavage JMJD2C leads to accumulated levels of H3K9 methylation and enhanced ability of repair of DNA DSB (double-strand breaks) [87]. Thus, these patients carrying only cleavable JMJD2C are resistant to chemotherapy and radiation therapy, which should be considered seriously in clinical practice.

## PERSPECTIVE

In summary, a number of evidences have suggested the association between JMJD2C protein and various tumors. Many efforts are underway or were already undertaken to add weight to prove JMJD2C protein is the indeed driver of tumorigenesis as well as to design specific JMJD2C inhibitors. Several types of JMJD2C inhibitors have been identified in the past few years, however, JMJD2C protein faces a variety of difficulties before acting as anticancer targets, and any kind of anticancer drugs require rigorous preclinical testing before practical application. For instance, mouse models should be used as invaluable tools for testing their effects *in vivo*, avoiding the side effects as few as possible [88, 89].

In any case, JMJD2C appears to be intricately interacting with tumor cells, and the understanding of how these interactions take place and how to apply them to clinical practice requires more studies. The protein-methyltransferase (PMT) inhibitors have been progressed to Phase I clinical trials [90], which arousing passions of researchers in tumor fields for targeting oncogenic transcription factors. Although targeting JMJD2C histone demethylases has not yet in the process for standard clinical application, the selective inhibitors for JMJD2C are becoming the candidate anticancer agents, which also provide the tools for exploring the biological mechanisms of JMJD2C.
